# *Ad* *hoc* Setup of an Online Mental Health Self-Help Program During the COVID-19 Pandemic: Description of the Development and Implementation Processes and Analysis of Its Users’ and Usage Profiles

**DOI:** 10.3389/fpsyg.2022.853371

**Published:** 2022-07-20

**Authors:** Matyas Galffy, Carina Bichler, Thomas Mohr, Jonas Egeter, Leonie Neu, Maria Oberhammer, Birgit Högl, Eberhard A. Deisenhammer, Barbara Sperner-Unterweger, Katharina Hüfner

**Affiliations:** ^1^Department of Psychiatry, Psychotherapy, Psychosomatics and Medical Psychology, University Hospital of Psychiatry II, Innsbruck Medical University, Innsbruck, Austria; ^2^Department of Sport Science, University of Innsbruck, Innsbruck, Austria; ^3^Center for Cancer Research, Comprehensive Cancer Center, Medical University of Vienna, Vienna, Austria; ^4^Department of Psychiatry, Psychotherapy, Psychosomatics and Medical Psychology, University Hospital of Psychiatry I, Innsbruck Medical University, Innsbruck, Austria; ^5^Department of Neurology, Medical University of Innsbruck, Innsbruck, Austria

**Keywords:** self-help, resilience, depression, anxiety, e-health, COVID-19

## Abstract

**Background:**

The COVID-19 pandemic hit Austria in March 2020. This led to a considerable reduction in outpatient psychiatric therapies. People with mental disorders as well as with newly emerging mental health issues found themselves with very limited treatment options. Within only a few days our hospital set up an online mental health self-help program which went online in its first version on the first day of the lockdown in Austria. The process of this development and implementation process alongside with the user’s and usage data for the program are presented here.

**Methods:**

A small core team initiated the development of the program on a low-budget basis and using mostly freely available digital resources. The program had to be free of costs for its users and easy to navigate. Each self-help module contains a text description of the topic, a self-rating questionnaire and several psychoeducational 2–5 min videos. These videos explain, e.g., interactions of mental stress and the immune system or the vicious circle of anxiety. Additional videos provide easy to learn techniques like breathing and relaxation exercises.

**Results:**

We illustrate the implementation of this program following the replicating effective program (REP) model. We provide a detailed description of the implementation process starting from a simple website to a smartphone-based application with registered user area and instantaneous reporting of self-rating questionnaire results to users. The described process could be used as a model for the setup of similar programs in a very short time. As an indicator of acceptance, we report 46,100 unique video views and 3,937 completed questionnaires in the first year of use. The most accessed videos were those on anxiety, relaxation and resilience. Analysis of the sociodemographic user data indicate that they were mostly young (< 45 years; 59.7%), females (77.5%) and previously mentally healthy individuals (74.5%). An example of the collected psychometric questionnaire data over time is given.

**Conclusion:**

We show that it is possible to set up an online mental health self-help program *ad hoc* and without extensive prior planning, which enabled us to dynamically respond to a new situation. We are now planning on keeping the program active for a longer period of time to supplement and expand traditional treatment settings also outside the COVID-19 pandemic.

## Background

SARS-CoV-2 infections were first recognized in December 2019 in China and soon cases appeared all over the world leading to the classification as a “pandemic” by the WHO in March 2020 (WHO Directors Speech, 2020). The first cases in the state of Tyrol, Austria, were identified in February 2020 and a lockdown was imposed first in Tyrol on March 18th, 2020, and subsequently all over Austria. During this first lockdown many treatment options for individuals living with a mental disorder came to a hold. Psychotherapeutic treatments and counseling options were paused and daycare or adjunct treatment options such as occupational therapy were no longer available. Patients without life-threatening diseases, i.e., most psychiatric and psychosomatic patients were even discharged from hospital to minimize risk of transmission within the healthcare structure and make room for SARS-CoV-2-infected individuals. Treatments at the psychiatric and psychosomatic outpatient and inpatient departments were reduced to emergency contacts all over the country, which were delivered *via* telephone whenever possible. During this time, however, we also observed an increase in contacts with individuals without previous mental disorders who were stressed by the pandemic or the quarantine situation. These contacts were referred to us from our emergency department and by help seeking individuals calling the department directly on the emergency telephone number. We faced the situation that fewer treatment options for help seeking individuals were contrasted by an increased demand for support.

Initial surveys in the context of the COVID-19 pandemic from the Hubei region in China have shown that almost 30% of the general population had moderate or severe anxiety symptoms and moderate to severe depressive symptoms (Wang et al., 2020). Similar numbers were found in other countries such as India at the beginning of the pandemic (Ahmad et al., 2020). The first European publications also delivered similar results. In Italy, which was one of the first countries in Europe to be affected by COVID-19, a study of 18,147 people showed that 37% were affected by post-traumatic stress symptoms, 17.3% by depression, 20.8% by anxiety, 7.3% by insomnia, 21.8% by high stress levels and 22.9% by adjustment disorders (Rossi et al., 2020). A study in Austria conducted in April 2020 (Pieh et al., 2020) showed comparable numbers with 21% of the respondents questioned suffering from symptoms of depression, 19% from anxiety and 16% from insomnia. Larger studies taking also control groups or longitudinal approaches into account have now confirmed a rise in stress-related mental disorders in individuals following COVID-19 (Taquet et al., 2021). In the general population a more differentiated picture is starting to evolve with a specific risk in individual societies and populations, during specific pandemic phases or for specific psychiatric symptoms. (Pierce et al., 2020; Oyenubi et al., 2022; Robinson et al., 2022)

We thus observed a pronounced mismatch between reduced help and treatment options for individuals with prior and newly occurring mental health problems and on the other hand treatment and counseling options reduced to only emergency settings. Therefore, we decided to establish an alternative intervention program and installed an online self-help tool with the subsequent evolution into a professional App covering also post-COVID problems. Digital online psychological treatments were also available before the COVID-19 crisis (da Fonseca et al., 2021) and have been shown to be effective (Andersson et al., 2019) but they were not widely used mainly due to technical limitations (Fant et al., 2021; Svendsen et al., 2021) and also to the fact that personal interaction and the therapeutic face-to-face relationship are viewed as a very important factor by many therapists (Chauhan and Jaiswal, 2017; Feijt et al., 2018). A recent review has underlined the effectiveness of self-guided interventions for anxiety, depression and stress-associated symptoms but also found reduced effectiveness compared to face-to face interventions (Fischer et al., 2020). E-health can be considered an emerging field at the intersection of medical informatics, public health and business, referring to health services and information delivered or enhanced through the Internet and related technologies (Eysenbach, 2001). Barriers for its implementation in routine care include clinician’s unwillingness to adopt e-health or problems with reimbursement; and while e-health services have proven useful during previous crisis, they are still not part of the routine repertoire of many healthcare systems (Smith et al., 2020). E-health is particularly interesting for circumstances and countries were face to face interventions are not possible or available and has been shown to be effective (Fu et al., 2020; Kazlauskas et al., 2020) in single trials and systematic reviews. Although exact numbers on a national or international level are scarce, the World Health Organization reported that in 2015 only a third of its member states indicated to have at least one program for technology-mediated mental health services (Hardiker and Grant, 2011; Organization, 2016). Increases in the use of e-health technology were taken up by North Atlantic Treaty Organization (NATO) as part of a disaster response plan (Doarn et al., 2018). Following the SARS pandemic there were initiatives in China to promote e-health services in general (Zhao et al., 2010). Nevertheless a needs-based and evidence-informed “whole system strategy” following the proposed e-Health Strategy Development Framework has not been widely implemented to date (Scott and Mars, 2013). However, the COVID-19 crisis has accelerated the development and research on e-mental health programs (Calkins, 2021; Drissi et al., 2021; Szlamka et al., 2021). At the time our online mental health self-help program was put into operation, to the best of our knowledge, there was no comparable e-health offer in Austria.

Here we describe the process of setting up such an online mental health self-help module within a very short time and initially without external support which might be a model for the provision of rapid mental health support during future crises.

## Methods and Setup

### Structure of Individual Modules of the Platform

**Figure 1:**
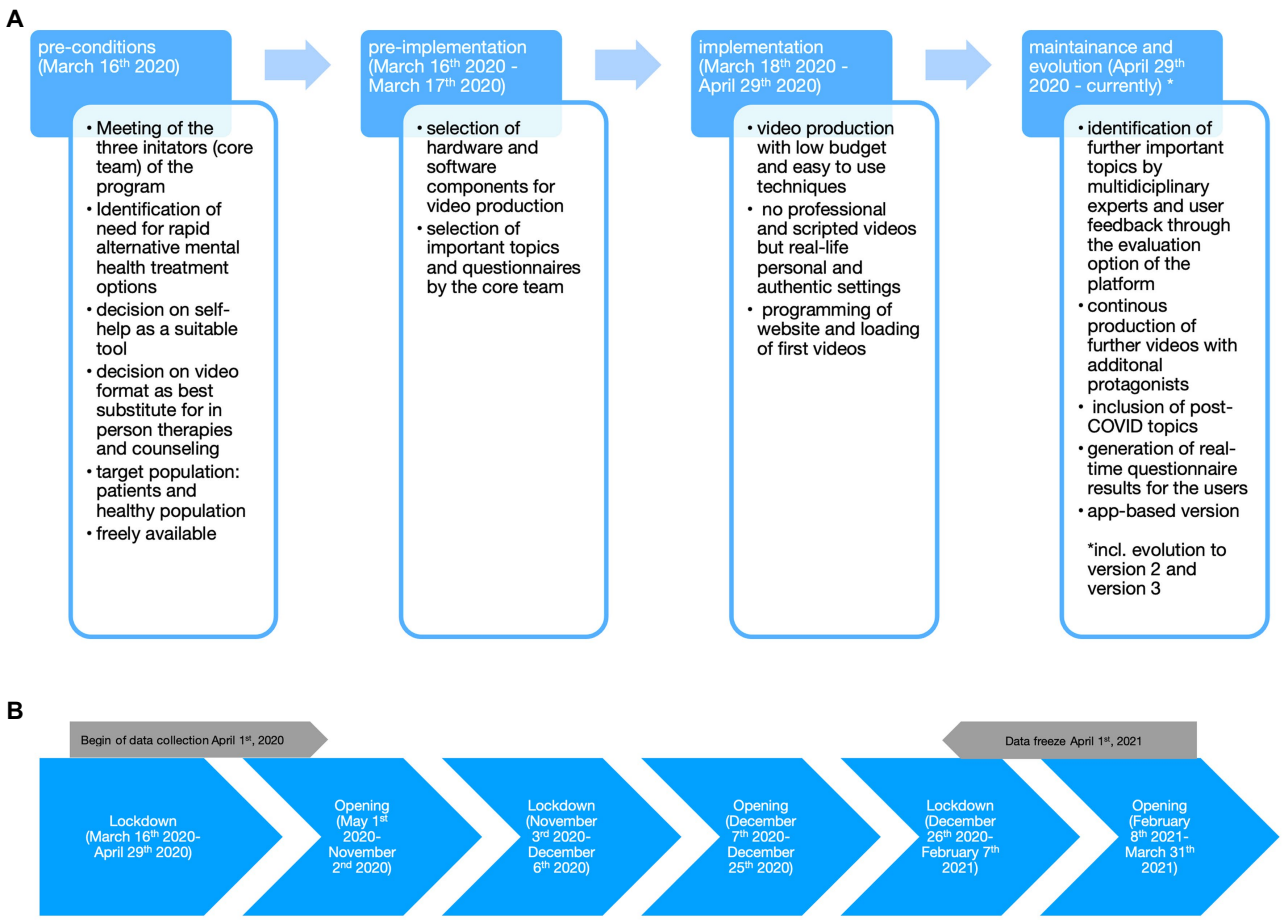
Components of the Replicating Effective Programs (REP) framework for the implementation of an online mental health self-help program and depiction of pandemic phases in Austria. **(A)** The different implementation phases are indicated (blue background) and the steps taken during the implementation of the current online mental health self-help program are described below in a general overview. **(B)** This figure shows the different lockdown and opening phases which took place during the first year of the pandemic in Austria.

**Figure 2:**
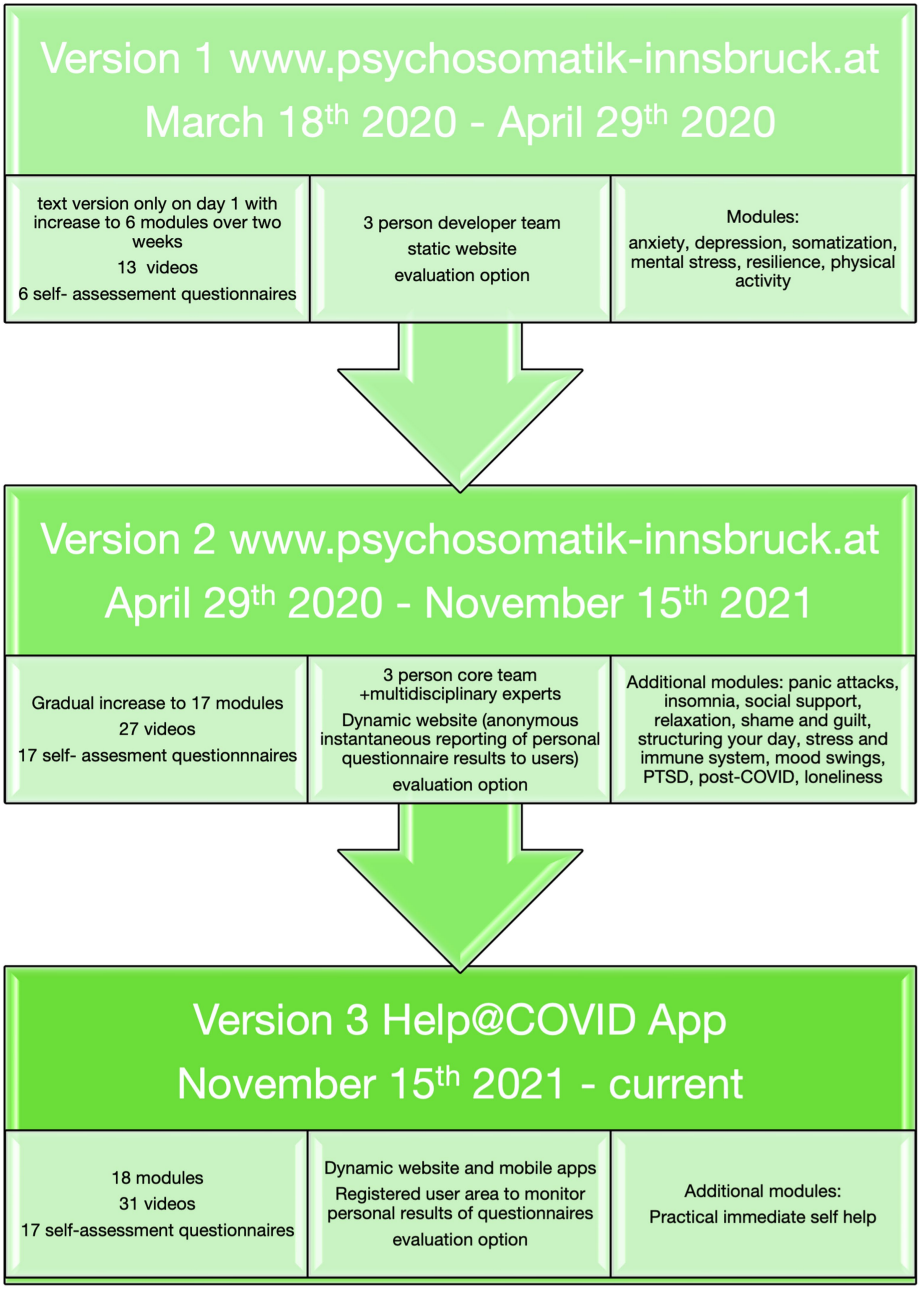
Timeline of project development. The individual questionnaires used for self-rating of mental health are described in the Supplemental Materials.

**Figure 3:**
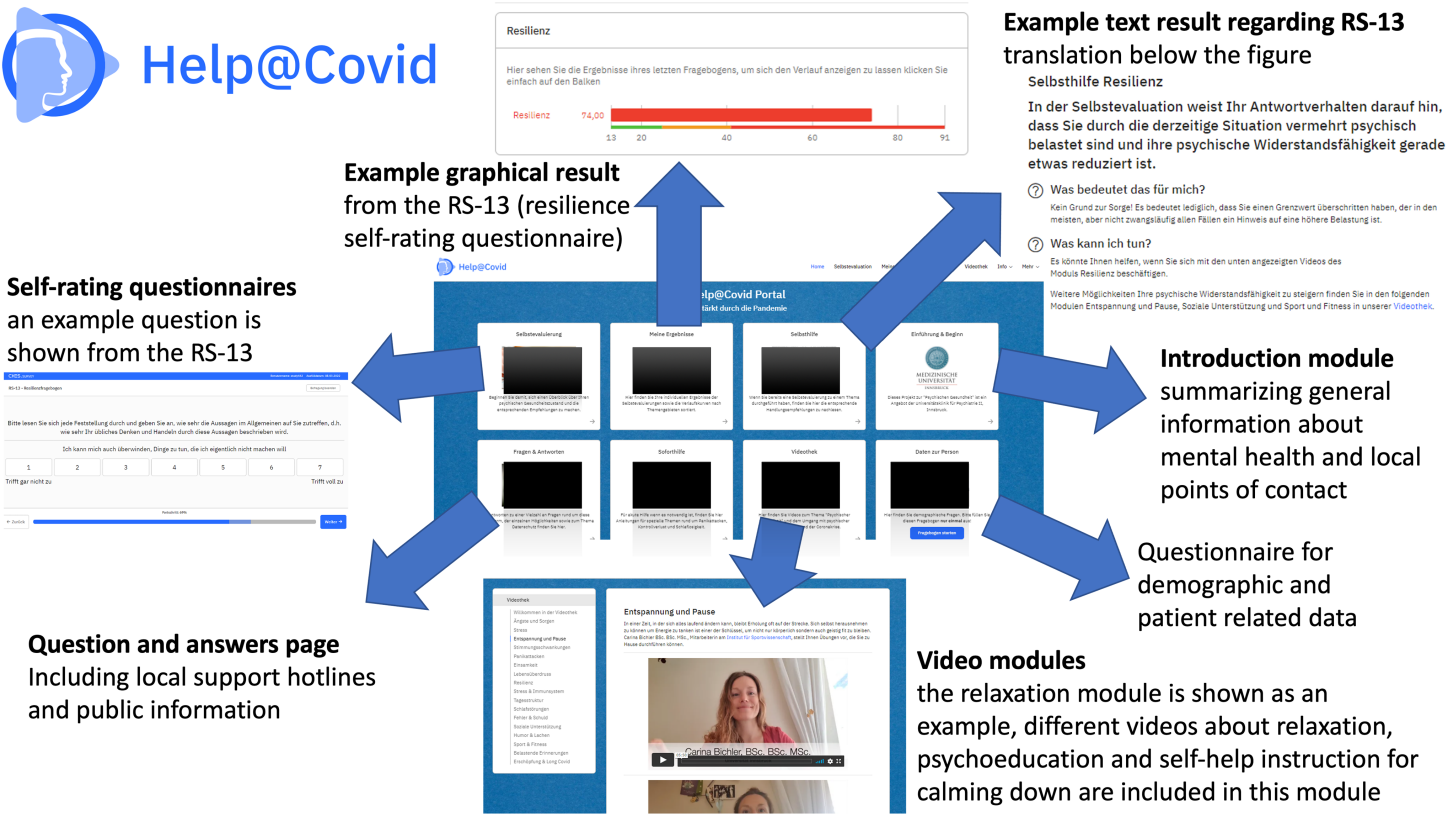
Overview over version 3 of the online self-help mental health platform. The different sections are illustrated with sample screenshots. The translation of the information supplied to explain the personal questionnaire scores in the section “self-help” is as follows: *“In the self-evaluation, your responses indicate that you are experiencing increased mental stress due to the current situation. Your resilience could also be reduced now. What does that mean for me? No reason to worry! It just means that your scores in the self-rating questionnaire have exceeded a threshold which can in some individuals be indicative of increased mental stress and burden. What can I do? You might find it helpful to watch the Resilience Module videos below. You can find more ways to increase your mental resilience in the following modules provided on this plaform: relaxation and breaks, social support and sport and fitness.”*Other parts are not translated in detail because the screenshots are only used to better illustrate the functioning of the online self-help mental health platform. RS-13, Resiliencescale 13 item version (Leppert et al., 2008).

The online platform was designed and developed in a modular manner to allow for flexibility and future adaptations. The overall layout varied slightly during the different evolution versions of the online mental health self-help program as well as between mobile and desktop versions (Figures 1, 2 show the different implementation stages, time periods and versions available from the start of the initiative up to now). Each module contains a short text description of the topic and the person presenting the video, the link to a self-rating questionnaire to assess the symptoms covered in the video, and one or several 2–5 min videos. These videos contain psychoeducative content such as, e.g., explaining interactions of mental stress and the immune system or the vicious circle of anxiety. Additional videos provide easy to learn psychotherapeutic techniques such as breathing and relaxation exercises or how to stop ruminating thoughts which mostly lend to cognitive behavioral therapy. The topics of these interventions focus around areas such as calming or creating a sense of self-efficacy, areas which have been identified to be important in the immediate response to mass traumatic events, although a pandemic is strictly speaking not considered a traumatic event in itself (Hobfoll et al., 2007). There is also a short welcome and introductory block, and all versions contained an index of the available topics. Figure 3 gives an overview over the platform design. While we encouraged users to fill out the self-assessment questionnaires prior to watching the video, this was entirely voluntary, and videos could also be watched without questionnaire completion. This was done to allow for high accessibility of the online mental health program.

### Technical Aspects

The online self-help platform consists of a Wordpress Content Management System (CMS)^1^ with Divi framework. Wordpress is a widespread free open-source content management system, Divi is a fee-based framework (design, functionality, and management of the system) from Elegant Themes Inc.,^2^ for which a corresponding license was available. In order to ensure fast access times of the videos, an external provider of video content (Vimeo LLC)^3^ was selected for better performance in case of high access. The first two versions of the platform have been financed, programed, and maintained on the initiative of the developing core team. They were held low budget and were developed with daily use items (commercial standard technical equipment). Equipment that was already available (e.g., laptop, webcam, and server) was used and only licenses had to be purchased (domain, video provider, and plugins), which meant that the total expenditure was around €300. The now available third version is financed with support of the Austria Wirtschaftsservice GmbH, Vienna, Austria. The online intervention platform can be accessed under the internet domain: https://www.psychosomatik-innsbruck.at.

The design of the platform was set up to be in a modular design with different thematic blocks so that it can be reduced or expanded as required. This procedure was chosen because of the lack of previous experience with online intervention programs and the uncertain development of the pandemic situation. This has the technical advantage that it is possible to add new content without affecting other topics during the update. The website was responsive and therefore optimized for mobile use.

Videos were recorded using a Logitech C920 pro conference webcam (Logitech International S.A., Apples, Switzerland) or Apple iPhone (Apple Inc., California, United States) connected to an Apple Macbook Pro and were edited with Apple iMovie. The media department of the hospital also recorded some videos in its studio and helped with post-processing of the videos in case of sound quality problems. The questionnaires were integrated into the website using independent programming by one of the authors (MG). Programming in the programming languages PHP (PHP: Hypertext Preprocessor) and JavaScript as well as adjustments in CSS (Cascading Style Sheets) were used. In addition, the results of the questionnaires were transferred to an Structured Query Language (SQL) database for storage and later evaluation. The output of the instantaneous reporting of personal questionnaire results to users in version 2 was done by the Wordpress plugin “WP-Testing,” a commercial plugin programed by Alexander Ustimenko (Wordpress.org, 2021). In version 3 and in the App, the instantaneous reporting of personal questionnaire results to users is created using the CHES system provided by ESD—Evaluation Software Development GmbH, Innsbruck, Austria, a company specializing on patient reported outcomes in clinical practice.

### Questionnaires

The use of questionnaire and usage data for research was approved by the ethics committee of Innsbruck Medical University (ethics vote EK1252/2020). Sociodemographic data including age, gender, work and quarantine situation and COVID-19 related questions such as whether the individual had previously been SARS-CoV-2 positive or in close contact with an individual who tested positive constituted the sociodemographic and clinical questionnaire. An overview of the 17 self-rating questionnaires used for mental health assessment can be found in the supplementary materials. The questionnaires were selected to cover the most common psychiatric symptoms, have some connection with the topic covered in the video and be freely available for online use. The depression anxiety stress scale (DASS) was used to measure anxiety, depression, and stress (Nilges and Essau, 2015) in all versions of the online self-help platform and data on depression and anxiety are reported in the manuscript as an example of collected questionnaire data. A button to evaluate the online mental health self-help platform is available at the bottom of the screen and allowed for a free text input.

### Data Privacy

In the first two website versions the entering of data into the self-assessment questionnaires was anonymous, it was, however, possible and encouraged to enter a personal code to allow for a linking of the sociodemographic questionnaire with the psychometric questionnaires. This was done at the user’s discretion and by protecting anonymity. From version 3 onwards users were able to create a user account and enter a protected area. Besides the credentials which are randomly generated the App stores no information about the user. When questionnaires are submitted or results are retrieved, all communication is conducted with a secure https-encrypted internet connection using the credentials only.

### Data Analysis

Data analysis for website and video access as well as for the evaluation of questionnaires was done from a data freeze referring to the data collected for 1 year, i.e., April 1st 2020 (date the first version of the website was in full operation)–April 1st 2021 (data freeze after 1 year). Data collection is, however, still ongoing to date. The different time phases analyzed were lockdown 1 [March 18th 2020 (data collection started April 1st 2020)–April 29th 2020], opening 1 (May 1st 2020–November 2nd 2020), lockdown 2 (November 3rd 2020–December 6th 2020), opening 2 (December 7th 2020–December 25th 2020), lockdown 3 (December 26th 2020–February 7th 2021) and opening 3 (February 8th 2021–April 1st 2021), see also Figure 1.

The video access data with statistical preparation beforehand from the analysis module by the streaming service provider Vimeo^4^ was exported into MS Excel^®^ and further statistically evaluated. The data show the total views of the individual videos and, depending on the individual data protection settings, also the views that can be clearly assigned to a device. Only accesses from Austria and *via* the main domain of the platform were evaluated. Other accesses from different top-level domains and countries were dismissed to restrict the analysis to the situation in Austria. It can be assumed that according to our selected security settings that other top-level domain accesses were through bots/machines and not by human users.

For statistical data analysis of the psychometric questionnaires’ regression analyses were performed on those datasets which could be linked to the sociodemographic data by participant code. The following variables were included to assess their influence on questionnaire scores: gender age, current physical illness, current mental illness, recovered from COVID-19 disease, close contact to a SARS-CoV-2 positive individual, number of people in household and the number of hours per day at home were used. Prior to analysis, data were checked for homoscedasticity using the Breusch-Pagan and the non-consistent error variance (NCV) test and the absence of multicollinearity.

Comparison of questionnaire data concerning the different pandemic phases was done using Wilcoxon test and corrections for multiple comparisons were performed using Benjamini Hochberg correction (Benjamini and Hochberg, 1995). The software GNU R version 4.1 (Team, 2021) was used for analysis and *p* < 0.05 considered significant in all analyses. For the violin-plots data are given as median with interquartile ranges (IQR).

## Results

### Time Course and Stages of Setup of the Online Interventional Platform

The online platform went online on the day the quarantine was imposed upon Tyrol on March 18th, 2020, and has since gone through multiple steps of evolution. The setup of the platform followed the Replicating Effective Programs (REP; Kilbourne et al., 2007) which consists of the following steps: pre-conditions (e.g., identifying need, target population, and suitable intervention), pre-implementation (e.g., intervention packaging and community input), implementation (e.g., package dissemination, training, technical assistance, and evaluation), and maintenance and evolution (e.g., preparing the intervention for sustainability). The REP process is shown in Figures 1A,B shows the pandemic phases (lockdowns and openings faced in Austria).

Approximately 1 week prior to the first launch a brainstorming meeting between the three initiators (MG, KH, and BU) took place to develop a pragmatic implementation strategy on the basis of the REP. This core team consisted to the department director, a consultant psychiatrist and an assistant doctor. The key objective was an immediate, cost-free and rapid development and access for patients and previously healthy individuals during the impending lockdown. This was deemed critical because of the increasing amount of telephone contacts with our department. After briefly considering possible e-health applications, we opted for a video-based unguided approach. In such, tool users cannot exchange information or interact with doctors or therapists but receive standardized, non-individually customized content. The core team opted for videos instead of a primarily text-based offer because this would best substitute for the treatment and counseling options which had been put on hold due to the pandemic. We also discussed other formats such as the use of chatbots (dismissed due to the preparation time and lack of experience within our team) or therapeutic fact-to-face contact (dismissed since human resources would have been tied up and thus not feasible on a large-scale during crisis times). The advantage of the selected approach is the higher number of people that can be reached with an unguided one way offer and the possibility for rapid development. With the online self-help program in place mental health providers could counsel clients and patients over the phone and refer them to the website for further information, video instructions and repeated viewing of information to gain safety and stability. In addition, the telephone line was kept free for emergency assistance.

Psychoeducative content and interventions from cognitive behavioral therapy were primarily used for the videos, as these are manual-based and the most reproducible. Proposed interventions adhered to evidence based treatment guidelines ((DeGPT) DGfPeV, 2020; Deutsche Gesellschaft für Psychiatrie und Psychotherapie (DGPPN), V. PuNe, 2020). The *ad hoc* setup was implemented by the core team within the first 3 days was then further developed and evolved subsequently to version 2 (Figures 1A, 2). Evaluation by users and multidisciplinary experts led to the expansion of the range of modules (videos and subject areas). User feedback provided *via* the evaluation option that the platform is slow and crashes on old devices supported the decision to further evolve the online mental health self-help platform into an App (with server-side content processing) in version 3 (Figure 2). Details of the individual versions can be found below. For a graphical overview of the platform see Figure 3.

#### First Version

On March 18th, 2020, a simple text-based website with tips for self-help in case of acute psychological distress as well as telephone numbers for various local and state COVID-related hotlines went into operation. Twelve days later (April 1st, 2020) the fully operational first version with online self-evaluation questionnaires and six mental health topics covered by text and video were published (Figure 2). It contained videos with a focus on psychoeducation and easy to learn self-help intervention as well as an evaluation option to improve the online mental health self-help program. The evaluation option was integrated because initially only the clinical experiences of the core team in exchange with patients from our hospital and data from the literature on past epidemics were considered when selecting the topics. In the evaluation option, users could describe in an open text field how they would rate the online mental health self-help program and what they would improve. The questionnaires were introduced in order to be able to monitor the psychosocial health status of the users and to adapt the content of the platform promptly since in this first version reporting back questionnaire results to participants was not possible.

#### Second Version

The major change from version one to version two was that the website was now dynamic, i.e., after completion of the self-assessment questionnaire related to the chosen mental health topic instantaneous reporting of personal questionnaire results to users was performed. Results reported to users indicated whether the score was high—medium—low (depending on questionnaire cut-offs), and what a potential meaning of this score could be. The focus was on indicative and non-diagnostic responses since the information is based on self-reports from the user and direct contact with professionals for verification of a potential diagnosis is not (yet) possible *via* the platform. In case of increased values for suicidality, a separate option for contacting professionals by telephone was offered. Videos were suggested to the participants according to their score on the respective questionnaire (Figure 2). At this point, with the increasing number of videos, the positive evaluation by users, and the positive reception in the local media [two local (Tyrolean) newspaper reports, published April 30th, 2020, one local TV report, aired on April 20th, 2020, one Austrian-wide newspaper report, published November 10th, 2020], more colleagues were willing to contribute videos. New videos were suggested either by the multidisciplinary experts from our hospital or by patients *via* the evaluation button. At this stage we also received encouraging feedback not only from the users themselves but also from colleagues from other medical disciplines (i.e., emergency medicine and internal medicine) so that the website including the QR code for access was posted at several strategical points around the hospital such as emergency and outpatient waiting areas. Examples of users’ evaluation given through the website included (English translation of German quotes): “Thank you for your care! I found help and information that was interesting to me!”; “I think it’s a great offer and it’s structured very clearly.”; “Great initiative. There is little reluctance or fear to try out the offer. Thank you!” and “Keep advice more specific, exercises, details for support in acute crises,” which has led to the development of immediate self-help module over time. The feedback could be given through a custom-developed questionnaire, which was embedded in the platform like the other questionnaires. Over all 48 feedbacks were submitted.

#### Third Version

Due to the persistently high number of mobile accesses (57% on mobile phones, tablet-computers), the focus of the further development was placed on mobile users. A further evolution was also necessary since, due to the one-pager design which displayed all modules and videos on one page, loading times increased as the content expanded, and browsers repeatedly crashed on old mobile devices. Also, the developers and users expressed the wish that results of questionnaires could be stored and compared to previous values to be able to detect improvements or deteriorations in mental health status over time. Therefore, the conversion to a mobile-friendly version including Apps for mobile devices was initiated. On November 15th, 2021, version 3 was published, and Apps in the Apple iOS Store and Google Play Store were released shortly thereafter (see Supplemental Materials for QR codes). The previously existing platform was also adapted for better user-friendliness and to the design of the Apps. It is now possible to enter a protected, registered user area where individuals can view the results of the questionnaires over time and share them with their healthcare providers in case they wish to (Figure 3).

### Analysis of Video Access Data

**Figure 4:**
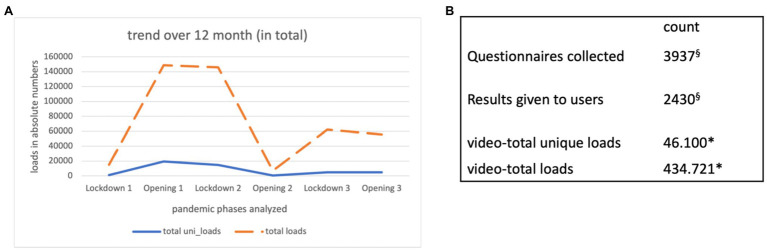
QR codes for App download and website access data collected during the first year of operation of the fully functional website. **(A)** The different time phases analyzed were lockdown 1 (March 18th, 2020 (data collection started April 1st 2020)–April 29th 2020), opening 1 (May 1st 2020–November 2nd 2020), lockdown 2 (November 3rd 2020–December 6th 2020), opening 2 (December 7th 2020–December 25th 2020), lockdown 3 (December 26th 2020–February 7th 2021) and opening 3 (February 8th 2021–April 1st 2021). Lockdown and opening phases refer to the situation in Austria. The number of total loads contains unique and also repeated loads or views that cannot be assigned (e.g., due to data protection settings of the user-device). Total_uni loads are the unique loads used to count unambiguous individual views, i.e., how many clearly distinguishable devices have played the video. **(B)** Shows the total numbers for the website statistics ^*^only the following views were counted: from Austria—only *via* main URL psychosomatik-innsbruck.at; The actual numbers due to views from other countries/domains (e.g., from Germany) could be higher. ^§^The difference results from the fact that in the first version there was no instantaneous reporting of personal results to users and that the sociodemographic questionnaire did not result in any user feedback.

When analyzing video access data, a distinction was made between total loads and unique loads (Figures 4A,B). Overall, we observed 46,100 unique video loads over all modules and 434,721 total loads (Figures 4A,B). The exact distribution of loads over the first year (April 1st, 2020–April 1st, 2021), are shown in Figure 4A. Access data were associated not only with the pandemic phases but also media presence of the development core team. There was a rapid increase in views during phase 1 (lockdown 1) as well as a decrease in phase 4 (opening phase in summer 2020). The ratio of unique loads to total loads is roughly 1:10 in phases 1–4 and increases subsequently, which could indicate repetitive views of videos by the same users but could also be related to the increase of available videos and publicity. Considering the expansion of the modules from six modules in version 1–17 modules in version 2, the evaluation results shows that the top five most accessed modules over the first year were “anxiety,” “mental stress,” “mood and mood swings,” “panic attacks,” and “resilience,” the least requested topics were “guilt,” “humor” and “social support” (in order from most to least accessed).

### Sociodemographic User Data

In total, 870 sociodemographic questionnaires were available and after cleaning the entries (38 entries had to be excluded due to unclear/missing data) 832 datasets remained. Sociodemographic data of users are presented in Table 1 in short to illustrate the user profile of our self-help mental health intervention program.

**Table 1:** Sociodemographic data collected *via* the website in the first year.

Socioemographic data of users (*n* = 832)		
Current physical illness	Yes	189 (22.7%)
	No	643 (77.3%)
Sex	Male	187 (22.5%)
	Female	645 (77.5%)
Age groups	<18	52 (6.3%)
	18–45	497 (5.7%)
	46–65	246 (29.6%)
	65+ years	37 (4.4%)
	<6	70 (8.4%)
	6–12	159 (19.1%)
	12–18	197 (23.7%)
Hours spent at home per day	18–24	406 (48.8%)
	Alone	232 (27.9%)
	2	293 (35.2%)
	3	139 (16.7%)
People in same household	4+	168 (20.2%)
Current mental illnes	Yes	212 (25.5%)
	No	620 (74.5%)
SARS-CoV-2 positive individuals in close contacts	Yes	185 (22.2%)
	No	647 (77.8%)

### Evaluation of Sample Questionnaire Data

**Figure 5:**
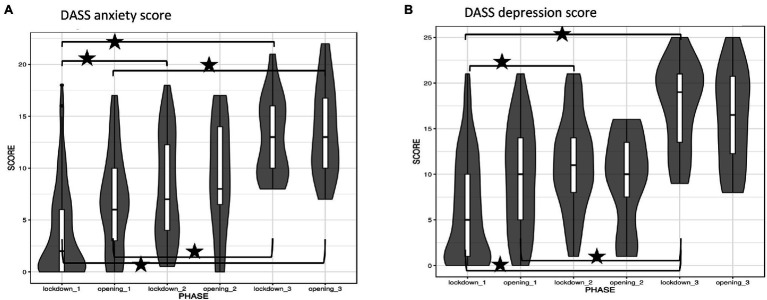
Example of psychometric self—rating questionnaires collected by the online mental health self-help platform. Data are shown as median and 25% quantile range. Significant differences between pandemic phases are indicated by asterisks (*p* < 0.01 adjusted for multiple comparisons). **(A)** DASS, depression anxiety stress scale—anxiety score. **(B)** DASS, depression score.

We selected one questionnaire from our platform to present exemplary results: the Depression Anxiety Stress Scale (DASS) with two subscales anxiety and depression. These two subscales were selected because they had been collected right from the first version of the intervention tool and these symptoms have been found to be important during the COVID-19 pandemic. The results were checked for internal consistency using Cronbach’s alpha and was high (Cronbach’s alpha for DASS: 0.962; Figures 5A,B). A total of 382 questionnaires were available for analysis. In the anxiety sub-subscale 190 (49.7%) showed increased values above the cut off (for anxiety >6 points) and in the depression sub-scale (cut off for depression >10 points) 172 participants (45%). The regression analyzes were run separately for the two subscales. For the anxiety sub-scale (215 questionnaires could be linked to the sociodemographic variables by code), there was a significant association of current mental illness (*β* = 3.5066, *p* < 0.001) as well as COVID-19 disease or SARS-CoV-2 positive individuals in their close contacts (*β* = 2.7457, *p* = 0.003) with higher anxiety levels. No effects of gender, current physical disease, age, people in same household, and hours spent at home per day was found. The adjusted *r*-squared was 0.1063. The depression sub-scale (215 questionnaires could be linked to the sociodemographics by code) also showed a significant impact of current mental illnesses (*β* = 3.8284, *p* < 0.01), female gender (*β* = −2.3789, *p* = 0.014) and younger age (*β* = −1.3966, *p* = 0.034) leading to higher depression scores. The adjusted *r*-squared was 0.1194. Acute physical disease, people in household, the hours spent at home per day and COVID-19 disease or SARS-CoV-2 positive individuals in their close contacts showed no effect.

## Discussion

We describe the rapid and low budget development and implementation of a mental health self-help online intervention program, following the principles of the REP. We highlight advantages of our approach and also the hurdles faced in the process by our team without extensive prior experience in the setup of e-health interventions. Our aim was to provide reproducible instructions. The main strength of this project was the short response time at which it was realized which was only possible by keeping the core development team small and using standard technical and open-source equipment. An additional strength is the low-threshold for using the online mental health self-help program and the high number of help-seeking individuals that can be reached. This was achieved through a video-based, unguided approach in which users were provided with videos for various mental health-related topics.

The psychoeducative videos and interventions were based mainly on cognitive behavioral therapy which have shown effects in reducing stress-associated symptoms (Fava, 2007) and are part of the evidence based treatment guidelines for anxiety disorders (Borza, 2017), somatic symptoms disorders (Orzechowska et al., 2021) or depression (Deutsche Gesellschaft für Psychiatrie und Psychotherapie (DGPPN), V. PuNe, 2020). Relaxation exercises especially related to breathing were based on existing evidence linking slow breathing with psychophysical changes and increased wellbeing (Zaccaro et al., 2018). Physical activity was recommended according to WHO standard and treatment guidelines (Bull et al., 2020) which now also emphasize the role of physical activity in mental health. Similar interventions as the ones we provided have been shown in a recent review to be effective for the self-guided treatment in depression or anxiety (Fischer et al., 2020; Zhang et al., 2021) and potentially useful during the COVID-19 pandemic.

The process of digitalizing mental health treatment during the COVID-19 pandemic has been taken on in many countries all over the world, which is also mirrored in corresponding publications (Liu et al., 2020; Rossi et al., 2020; Drissi et al., 2021; Farris et al., 2021; Szlamka et al., 2021), and is proving to be an effective way for other types of treatment. The available literature describe different projects and approaches, from individual and smaller scale programs (Zhang et al., 2020; Szlamka et al., 2021) to government initiated widely broadcasted ones (He et al., 2020). In general, the use of online mental health services over the past 1.5 years has facilitated the development of public emergency interventions (Madanian et al., 2020; Smith et al., 2020). It has also opened new treatment avenues such as artificial intelligence identifying individuals at risk of suicide by analyzing social media data, which could eventually improve the quality and effectiveness of emergency interventions (Liu et al., 2020). But hurdles associated with the delivery of online mental health services have also come to attention and include legal questions and questions related to the practical administration of digital tools (Situmorang, 2020; Szlamka et al., 2021). In our case the high number of accesses lead to an increased overloading (long loading times, crashes) which had to be solved by evolution of the website into an App and a corresponding server structure. Previous experience (Smith et al., 2020) has shown that many e-health programs can fail in crisis situations because they are not put into operation on time, the technical options are not adequate or they are not well received by the people they offer was designed for. Insufficient funding and questions related to reimbursement, the organization of the healthcare system itself and reluctance of the involved healthcare personnel are further barriers (Smith et al., 2020).

The high numbers of female users and younger individuals in our sociodemographic database indicate that the online self-help program is used mainly by people who are identified as a risk group of higher mental stress in the COVID-19 crisis in Austria (Pieh et al., 2020). This underlines the usefulness of offering online mental health self-help especially if the fact that our program is freely available is also considered (Shigekawa et al., 2018). The online mental health self-help program was also used by many previously healthy individuals, suggesting an increased interest in mental health topics and possibly a higher burden of mental distress in the general population during the pandemic. The high number of accesses in early phases of the pandemic corresponds to literature showing an elevated burden of mental health symptoms especially in the early phase of the pandemic (Robinson et al., 2022). However, the observed usage data were most likely also influenced by media coverage and the presence of local publicity at the authors´ hospital. There is also the possibility that governmental regulations such as lockdowns had an impact on usage data since there is this has shown an albeit small, effect on mental wellbeing (Prati and Mancini, 2021). We observed high number of mobile accesses (57% of access per mobile phones and/or tablet-computers) which corresponds to the experiences of other researchers (Fiol-DeRoque et al., 2021). While our usage data suggest that individuals performed multiple views of our online mental health self-help offer, we cannot make any claims as to adherence which is generally considered a problem in online interventions. Several theoretical models have been developed to address user adherence in e-health interventions (Ryan et al., 2018). We focused on improving adherence and tried to include lessons from other experiences, in particular the need to keep health messages simple, tailored to the individual and repeated frequently on demand (Price, 2016).

The analysis of our questionnaire data on mental health shows a worsening of mental health in the individual questionnaires from lockdown three onwards. However, our data are not true longitudinal data because they were not collected from the same sample over time. The longitudinal data available in the literature up to now show a worsening of mental health in the general population during the initial phases of the pandemic with especially large effects for depressive symptoms Our data rather show a worsening of symptoms of anxiety and depression over the first year of the pandemic, we can however make no claims as to whether this is a true deterioration of mental health status in the general population or whether the users of our online mental health self-help tool shifted to include more individuals with mental disorders over time since we started regularly recommending the tool as an add on treatment to our patients and are still doing so. Persisting psychological distress has been found at 2 months following self-isolation or quarantine especially in younger individuals and females (Maggi et al., 2021), comparable to our results in the initial phase of the pandemic in Austria (Pierce et al., 2020).

### Limitations

Our experience of setting up an online self-help mental health program is quite descriptive, and not generalizable to all other countries/settings. Also, the displayed data refer to different versions of the platform and different phases of the pandemic and we acknowledge that the recorded psychometric data can be biased since they were not collected at random and cannot be matched with the sociodemographic data in many cases. However, the principal components such as the high accessibility or modular successive buildup are probably useful for most settings and the detailed description allows for modifications and adaptations.

While the setup of our online mental health self-health platform was in many ways successful we agree with authoritative experts on e-health that it would create greater benefits in the long term to implement e-health options and programs proactively rather than reactively to help with the everyday and emergency challenges in healthcare (Smith et al., 2020). Through the approach we chose we tried to overcome some of the typical obstacles observed in e-health mental health programs such as not dealing with topics users care most about, not respecting privacy, not being considered as trustworthy or being unhelpful in emergencies (Torous et al., 2018).

### Lessons learned

- The use of “standard” technical equipment and open-source resources made it possible to keep the initial development and implementation costs low.
- Keeping the development core team small but nevertheless interacting with users and multidisciplinary experts proved to be valuable to customize the online mental health self-help program to the need to its users.
- Providing interventions which we use in routine care at our department in a self-help format without scripted texts or staged environments helped to keep them close to the clinical care formats individuals are used to and might have helped to build trust in the online mental health self-help platform.
- Additionally, we found that implementing an online mental health self-help program which can then subsequently be further developed was very helpful in practice. This allows to dynamically respond to usage data (e.g., optimize for mobile accesses or increased trafficking).

## Conclusion

We show that it is possible to set up an online mental health self-help program *ad hoc* and without extensive prior planning which enabled us to dynamically respond to a new situation.

Self-help programs can strengthen self-efficacy which is one of their main advantages. According to Bandura’s theory of self-efficacy individuals will only undertake activities or persevere in the face of difficulties if they believe they can produce desired effects by their actions (Bandura and Adams, 2005). The platform expands treatment options for mental disorders and subsyndromal psychological distress and might be a valuable addition to standard face-to-face mental health treatments also once the pandemic subsides.

## Data Availability Statement

The original contributions presented in the study are included in the article/Supplementary Material, further inquiries can be directed to the corresponding author.

## Ethics Statement

The studies involving human participants were reviewed and approved by the ethical review committee of Medical University Innsbruck, Austria. Written informed consent was not provided because of retrospective evaluation of existing data. Permission was obtained from the individual(s) for the publication of any potentially identifiable images included in this article.

## Author Contributions

MG, KH, and BS-U: conceptualization. TM, MG, and KH: formal analysis. MG, KH, BS-U, CB, JE, LN, MO, BH, and ED: resources. MG and KH: writing—original draft, visualization, and funding acquisition. CB, JE, LN, MO, BH, and ED: writing—review and edition. All authors contributed to the article and approved the submitted version.

## Funding

Development of version 3 of this project was funded by Austria Wirtschaftsservice GmbH, Vienna, Austria (grant number P2283018-TBX02). The funder was not involved in the study design, collection, analysis, interpretation of data, the writing of this article, or the decision to submit it for publication.

## Conflict of Interest

The authors declare that the research was conducted in the absence of any commercial or financial relationships that could be construed as a potential conflict of interest.

The reviewer MC declared a shared affiliation with the author(s) CB to the handling editor at the time of review.

## Publisher’s Note

All claims expressed in this article are solely those of the authors and do not necessarily represent those of their affiliated organizations, or those of the publisher, the editors and the reviewers. Any product that may be evaluated in this article, or claim that may be made by its manufacturer, is not guaranteed or endorsed by the publisher.

## Footnotes

^1^ https://wordpress.org

^2^ http://elegantthemes.com

^3^ https://vimeo.com

^4^ http://vimeo.com
