# Supplementary material for: Ad hoc Setup of an Online Mental Health Self-Help Program During the COVID-19 Pandemic: Description of the Development and Implementation Processes and Analysis of Its Users’ and Usage Profiles
Source: Front Psychol. 2022 Jul 20;13:853371. doi: 10.3389/fpsyg.2022.853371 (PMC9350598; doi:10.3389/fpsyg.2022.853371)
Supplement: Supplementary file 1 [file Data_Sheet_1.PDF]

Questionnaires

In addition, the following questionnaires were used: the GAD-7 to measure anxiety (a subscale of the Patient Health Questionnaire PHQ-D) (1), the PHQ-9 to measure depressive symptoms (subscale of the PHQ-D) (1), the PSS-10 to measure mental stress (Perceived Stress Scale - 10) (2), the RS-13 to measure resilience (Resilience Scale - 13) (3), the IPAQ short form to measure physical activity in metabolic units (international physical activity questionnaire – short form) (4), the PHQ-D panic module to assess panic attacks (subscale of the patient health questionnaire PHQ-D) (1), the ISI to assess sleep (insomnia severity index) (5), the SWE to assess self-efficacy (Skala zur Allgemeinen Selbstwirksamkeitserwartung) (6), the EUROHIS-QOL-8 for quality of life (EUROHIS Quality of Life - 8, short form of WHOQOL-brev) (7), the SCI subscale for stress coping (Stress and Coping Inventory) (8), the SSGS to measure shame and guilt (State Shame and Guilt Scale) (9), the SCI subscale for mental stress (Stress and Coping Inventory) (8), the PHQ-D somatization module to assess somatization (subscale of the patient health questionnaire PHQ-D) (1), the DASS to measure anxiety, depression, stress (Depression Anxiety Stress Scale) (10), the IES-R to assess symptoms of posttraumatic stress disorder (Impact of Event Scale – revised) (11), the CFS to assess fatigue (Chalder Fatigue Scale) (12), and the UCLA 3 ILS for loneliness (UCLA 3 Item Loneliness Scale) (13).

QR Codes for mobile application

A

B

A) QR code for Apple iOS Store B) QR Code for Google PlayStore

1. Gräfe K, Zipfel S, Herzog W, Löwe B. Screening psychischer Störungen mit dem "Gesundheitsfragebogen für Patienten (PHQ-D)". Diagnostica. 2004;50(4):171-81.

2. Schneider EE, Schönfelder S, Domke-Wolf M, Wessa M. Measuring stress in clinical and nonclinical subjects using a German adaptation of the Perceived Stress Scale. International Journal of Clinical and Health Psychology. 2020;20.

3. K. Leppert BK, E. Brähler, B. Strauß. Die Resilienzskala (RS) - Überprüfung der Langform RS-25 und einer Kurzform RS-13. Klinische Diagnostik und Evaluation. 2008;2:226-43.

4. Lee PH, Macfarlane DJ, Lam TH, Stewart SM. Validity of the International Physical Activity Questionnaire Short Form (IPAQ-SF): a systematic review. Int J Behav Nutr Phys Act. 2011;8:115-.

5. Bastien C, Vallières A, Morin C. Validation of the Insomnia Severity Index (ISI) as an outcome measure for insomnia research. Sleep medicine. 2001;2:297-307.

6. Schwarzer RJ, M. SWE. Skala zur Allgemeinen Selbstwirksamkeitserwartung [Verfahrensdokumentation, Autorenbeschreibung und Fragebogen]. Leibniz-Institut für Psychologie (ZPID) Open Test Archive Trier: ZPID 2003.

7. Schmidt S, Mühlan H, Power M. The EUROHIS-QOL 8-item index: psychometric results of a cross-cultural field study. European Journal of Public Health. 2006;16(4):420-8.

8. Satow L. Stress- und Coping-Inventar (SCI) [PSYNDEX Tests-Nr. 9006508]. . Leibniz-Zentrum für Psychologische Information und Dokumentation (ZPID) 2012(Elektronisches Testarchiv).

9. Cavalera C, Pepe A, Zurloni V, Diana B, Realdon O. A short version of the state shame and guilt scale (SSGS-8). TPM - Testing. 2017;24:1-8.

10. Nilges P, Essau C. Die Depressions-Angst-Stress-Skalen. Der Schmerz. 2015;29(6):649-57.

11. Rosner R, Hagl M. Die revidierte Impact of Event-Skala (IES-R). Psychosomatik und Konsiliarpsychiatrie. 2008;2(4):240-3.

12. Morriss RK, Wearden AJ, Mullis R. Exploring the validity of the Chalder Fatigue scale in chronic fatigue syndrome. J Psychosom Res. 1998;45(5):411-7.

13. Hughes ME, Waite LJ, Hawkley LC, Cacioppo JT. A Short Scale for Measuring Loneliness in Large Surveys: Results From Two Population-Based Studies. Res Aging. 2004;26(6):655-72.
